# Visualizing the appearance and disappearance of the attractor of differentiation using Raman spectral imaging

**DOI:** 10.1038/srep11358

**Published:** 2015-06-16

**Authors:** Taro Ichimura, Liang-da Chiu, Katsumasa Fujita, Hiroaki Machiyama, Satoshi Kawata, Tomonobu M. Watanabe, Hideaki Fujita

**Affiliations:** 1 Laboratory for Comprehensive Bioimaging, Riken QBiC, 6-2-3 Furuedai, Suita, Osaka, Japan; 2 Department of Applied Physics, Osaka University, 2-1 Yamadaoka, Suita, Osaka, Japan; 3 WPI, Immunology Frontier Research Center, Osaka University, 1-3 Yamadaoka, Suita, Osaka, Japan; 4 Nanophotonics Laboratory, RIKEN, Wako, Saitama, Japan, 2-1 Hirosawa, Wako, Saitama, Japan

## Abstract

Using Raman spectral imaging, we visualized the cell state transition during differentiation and constructed hypothetical potential landscapes for attractors of cellular states on a state space composed of parameters related to the shape of the Raman spectra. As models of differentiation, we used the myogenic C2C12 cell line and mouse embryonic stem cells. Raman spectral imaging can validate the amounts and locations of multiple cellular components that describe the cell state such as proteins, nucleic acids, and lipids; thus, it can report the state of a single cell. Herein, we visualized the cell state transition during differentiation using Raman spectral imaging of cell nuclei in combination with principal component analysis. During differentiation, cell populations with a seemingly homogeneous cell state before differentiation showed heterogeneity at the early stage of differentiation. At later differentiation stages, the cells returned to a homogeneous cell state that was different from the undifferentiated state. Thus, Raman spectral imaging enables us to illustrate the disappearance and reappearance of an attractor in a differentiation landscape, where cells stochastically fluctuate between states at the early stage of differentiation.

Cell differentiation is a complex process controlled by gene expression cascades that result in cellular advancement to a stable, minimal-potential state. This process is often described as a ball rolling down a potential landscape. The idea was first proposed as Waddington’s epigenetic landscape^1^, and the theoretical framework has been organized in complex systems science^2^,^3^,^4^. Many attempts have been made to describe the potential landscape of differentiation in actual cells with observable parameters such as epigenetic changes or gene expression^5^,^6^,^7^,^8^. These studies have comprehensively examined a number of components in the system of interest, such as gene expression, and attempted to explain the behaviour of the system through the extracted components. To estimate the landscape structure, experimental parameters such as expression level and the positions of related proteins must be quantitatively estimated in a single cell. Such decomposition analyses are, however, currently difficult at the single-cell level. Thus, a novel technology is required to construct proper models. Herein, we propose a methodology that uses a set of simple parameters related to the Raman spectrum of a cell to understand the change in landscape structure during differentiation.

Raman scattering micro-spectroscopy is increasingly used in cell biology because it uniquely reports the number of molecular species without labeling^9^,^10^,^11^. Almost all biomolecules are Raman-active, including proteins, lipids, nucleic acids, and many other small molecules, and chemical information can be determined from a Raman spectrum of the biomolecules. In laser scanning Raman microscopy of cells, a Raman spectrum is, in principle, a superposition of the Raman spectra of all molecular species present within a focal spot. Recently, we showed that morphological analysis of the complicated Raman spectra from the cell nucleus can identify the cell state transition during embryonic stem cell (ESC) differentiation^12^. Intriguingly, we found that spontaneous ESC differentiation results in a homogeneous cell population and conducted further detailed investigation to interpret the phenomenon.

In this study, we aimed to establish a method to visualize the appearance and disappearance of attractors in the differentiation landscape using Raman spectroscopy. For this purpose, we used myogenic cell line C2C12 and ESCs as a model of cell differentiation. Myogenesis is a well-studied differentiation model in which expression of muscle-specific genes and fusion of myoblasts result in the formation of a multi-nucleated myotube with sarcomere structure and contractility. Furthermore, it is easy to identify the differentiation state by cellular morphology. We here demonstrate the procedure to elucidate the C2C12 differentiation landscape using Raman spectra and verify its applicability to the other cells using ESCs. This method is capable of describing the cell state transition in a single cell; thus, Raman spectral imaging is a valuable technique for investigating cell state without labelling or cell disruption.

## Results

### Raman imaging of C2C12 cells

At each stage of C2C12 differentiation, cell morphology changed: cells were wide spread with extending pseudopods (Day -3), cells becoming round and small (Day 0), cells starting to elongate (Day 3), and form tubular structures (Day 7) (Fig. S1A). Myogenin appeared on Day 1 and gradually increased until Day 7, while the level of myosin heavy chain (MHC) gradually increased from Day 3 and that of tropomyosin significantly increased from Day 5 (Fig. S1B). Immunofluorescence revealed huge variation among individual cells: some cells express large amount of myogenin or MHC at Day 3, whereas others show a negligible amount (Fig. S1C, Day 3). On Day 7, presence of MHC-negative cells which failed to fuse and form myotubes can be observed (Fig. S1C, Day 7). These pre-experimental data indicate the validity of cell state identification using cellular morphology and the importance of knowing cell states at the single-cell level for understanding the dynamics of cell state transition.

**Figure 1:**
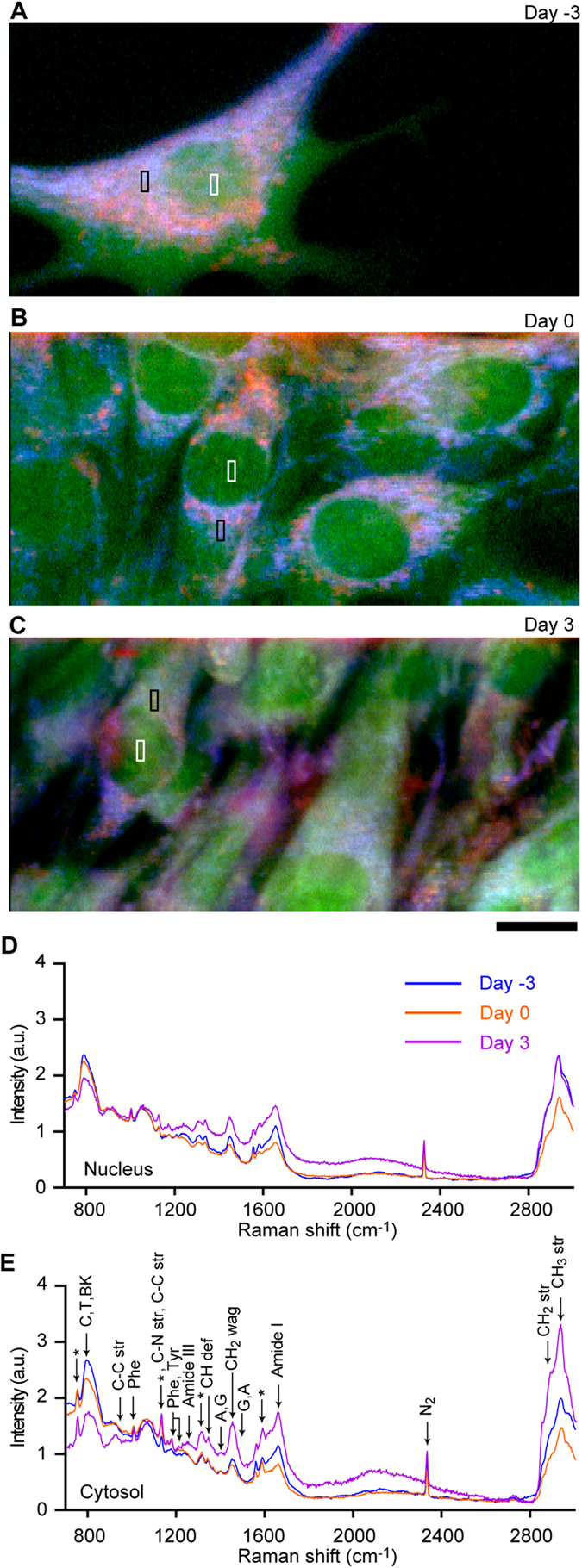
Raman images of C2C12 myoblasts at various stages of differentiation. (**A**–**C**) RGB reconstitution of C2C12 myoblasts before induction of differentiation (**A**), when cells became confluent (**B**), and three days after induction of differentiation (**C**). Raman peaks at 753 cm^−1^ (cytochrome C), 1004 cm^−1^ (proteins), and 2852 cm^−1^ (lipids) are mapped in blue, green, and red, respectively. Scale bar, 10 μm. (**D**,**E**) Representative Raman spectra from cell nucleus (**D**) and cytosol (**E**) at various stages of differentiation. The representative Raman spectra shown are the average of spectra in the rectangle regions in A–C. Area of each rectangles are 1.63 μm^2^ (49 pixels). Peak assignments: RP, ribose-phosphate; BK, backbone OPO of nucleic acids; str, stretching; wag, wagging; def, deformation^30^. Peaks characteristic to cytochrome C are indicated with asterisks.

Raman microscopy enables visualization of C2C12 cell morphology with the information about the molecular species that comprise the cell (Fig. 1). We allocated colours to the spectral peak intensities at 753 cm^−1^ (pyrrole ring breathing mode in cytochrome C; blue), 1004 cm^−1^ (CH_3_ stretching mode mainly in proteins; green), and 2852 cm^−1^ (CH_2_ stretching mode mainly in lipids; red) (Fig. 1A–C). On Day 3, we observed a broad distribution of cytochrome C in the cytosol (blue) and strong accumulation of lipid droplets (red). However, proteins were evenly distributed both in the cytosol and the nucleus under all conditions (green). Using the location information from the images (Fig. 1A–C), we compared the Raman spectra for the nucleus (Fig. 1D) and cytosol (Fig. 1E) at various stages of differentiation. The spectral shape changes in both locations upon differentiation but shows larger stage-dependent changes in the cytosol than in the nucleus. Significant increases in peaks corresponding to CH_2_ and CH_3_ stretching were observed on Day 3, indicating an increased amount of lipids in the cytosol. We decided to concentrate on the nucleus in this study to avoid the effects of auto-fluorescence and spectral diversity within the cytosol.

### Difference in spectral shape at various stages of differentiation

**Figure 2:**
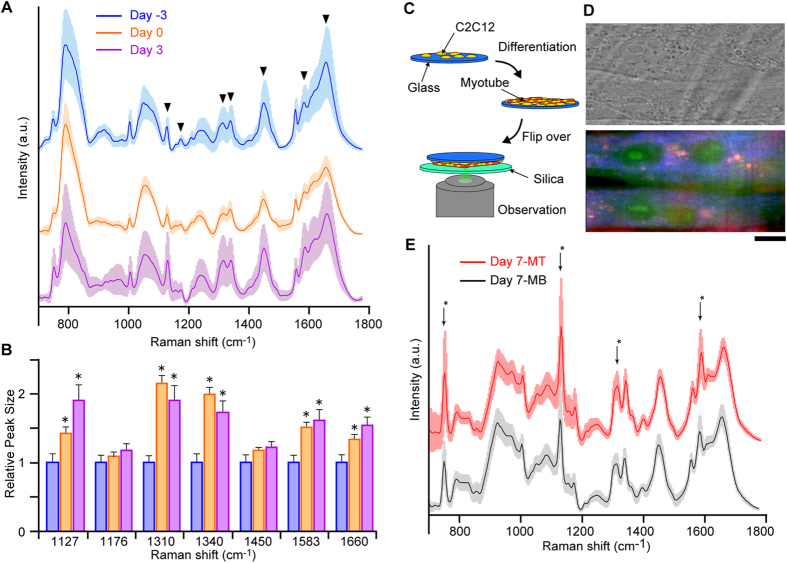
Comparison of Raman spectra at various stages of myogenesis. (**A**) Averaged Raman spectra of C2C12 myoblasts at Day -3 (blue), Day 0 (orange), and Day 3 (purple). The lower envelope, which was estimated by fourth-order polynomial fitting, was subtracted from all spectra to make the spectral differences clearer for comparison^31^. Solid lines are averaged spectra and faded ribbons of the same colour show SD. Spectra were obtained from 24 nucleus for Day -3, 61 for Day 0, and 42 for Day 3 myoblasts. For each nuclei, spectra was obtained from rectangle area consisting 2.70 μm^2^ (81 pixel). Data was obtained from three independent cell culture performed on different days. (**B**) Relative areas of Raman peaks indicated by inverted triangles in (**A**) at Day -3, Day 0 and Day 3. Each peak area was normalized to the peak at 1004 cm^−1^. Data are displayed relative to the Day -3. Asterisks show p < 0.05 compared with undifferentiated myoblasts. Error bar, SE. (**C**) Schematic illustration of the imaging procedure of C2C12 myotubes. Cells were first cultured on a borosilicate glass coverslip and differentiated into myotubes. For observation, the borosilicate glass coverslip was flipped over and placed on a silica coverslip to reduce the signal from the glass. (**D**) Bright field image (top) and Raman image (bottom) of myotubes. Colour coding is the same as in Fig. 1. Scale bar, 10 μm. (**E**) Averaged Raman spectra of the nuclei at the fingerprint region (700–1800 cm^−1^) of C2C12 myoblasts (Day 7-MB, black) that failed to fuse to myotubes and myotubes (Day 7-MT, red) that were successfully fused. Spectra were obtained from 53 nucleus for myotubes and 18 for myoblasts which fail to fuse to myotubes. For each nuclei, spectra was obtained from rectangle area consisting 2.70 μm^2^ (81 pixel). Data was obtained from three independent cell culture performed on different days. The significant difference in the spectral shape of myotubes is caused by the difference of the measurement geometry, shown in (**C**). Peaks characteristic to cytochrome C are indicated with asterisks.

To visually recognize the spectral changes in the fingerprint region acquired from the nucleus at various stages of differentiation, averaged spectra from 24 nucleus for Day -3, 61 nucleus for Day 0 and 42 nucleus for Day 3 myoblasts were calculated (Fig. 2A). Data were obtained from three independent cell culture performed on different days. The spectra were processed by subtracting the lower envelope. Spectral features are significantly different at each stage of differentiation, and increases in the levels of cytochrome C (1127 cm^−1^ and 1310 cm^−1^), nucleic acids (1340 cm^−1^) and proteins and lipids (1450 cm^−1^) were observed after differentiation. To quantify these changes, we calculated the size of the Raman peaks from the cell that are well separated from peaks originating from the silica substrate (Fig. 2B). The Raman peak areas were first normalized to the peak at 1004 cm^−1^ within each spectrum and further normalized to the peak size of undifferentiated cells. We chose peak at 1004 cm^−1^ for normalization because this band is breathing mode of phenylalanine in the protein which is commonly used for normalization because this band is not sensitive to conformational changes of protein. The peaks at 1310 cm^−1^ and 1340 cm^−1^ doubled in size when cells reached confluence even without induction of differentiation through reducing the serum concentration, indicating a change in cell state after reaching confluence. Initiation of differentiation by confluency is in good agreement with the previous findings that C2C12 cells initiate differentiation via N-cadherin-dependent signalling through cell-cell contacts on reaching confluency^13^,^14^,^15^. Other peaks show a gradual increase upon progression of differentiation, indicating that Raman peak size can be used to determine the extent of myogenesis. Thus, Raman spectra enable investigation of changes in the global chemical components of an individual single cell.

Progression of C2C12 differentiation leads to cell fusion and formation of multi-nucleated myotubes with contractility. It is of interest whether Raman spectra from myotubes differ from those of myoblasts; however, long-term differentiation of C2C12 on the silica substrate failed as they detached 4–5 days after induction of differentiation. This is possibly due to the tension generated by differentiating cells reported previously^16^. Furthermore, myotubes tend to position above undifferentiated myoblasts, increasing the difficulty of Raman observation. To overcome these difficulties, we cultured C2C12 myoblast cells on a gelatine-coated glass coverslip for 7 days so that most of the myoblast cells were differentiated into myotubes (Fig. 2C). The glass coverslip was then flipped and placed on a silica coverslip for observation of myotubes through the silica coverslip. This successfully enabled Raman observation of myotubes, showing the multi-nucleated tubular myotubes in the RGB reconstituted Raman image (Fig. 2D). The Raman image shows a strong signal from cytochrome C compared with surrounding cells that are not fused to form myotubes, indicating the well-developed mitochondria in differentiated myotubes.

Figure 2E shows averaged Raman spectra of the fingerprint region obtained from the nucleus of the myotubes (Day 7-MT; red) and from myoblasts that failed to fuse (Day 7-MB; black). Spectra were obtained from 53 nucleus for myotubes and 18 for myoblasts which fail to fuse to myotubes. Data was obtained from three independent cell culture performed on different days. The large differences in spectral features compared to myoblasts in Fig. 2A are due to the Raman signal from the glass coverslip. Small but clear differences were seen between the Raman spectra from myotubes and myoblasts, with stronger cytochrome C peaks in myotubes (Fig. S2). This result indicates that Raman spectra are different between undifferentiated myoblasts and myotubes. Thus, we were able to collect distinct Raman spectra corresponding to the progressing differentiation stages in myogenesis.

### Drawing the landscape of differentiation with principal component analysis

To describe the differences in Raman spectra at various stages of differentiation on a hypothetical potential landscape, we applied principal component analysis (PCA), a multivariate analysis method widely used for spectral analysis in which the spectra are decomposed into a linear combination of loading vectors after extracting the number of independent components. The loading vectors form an orthonormal coordinate system with the deduced dimension, and the scores represent the weight coefficients for the loading vectors^17^, which we adopt as state parameters to quantitatively express the state of cells.

**Figure 3:**
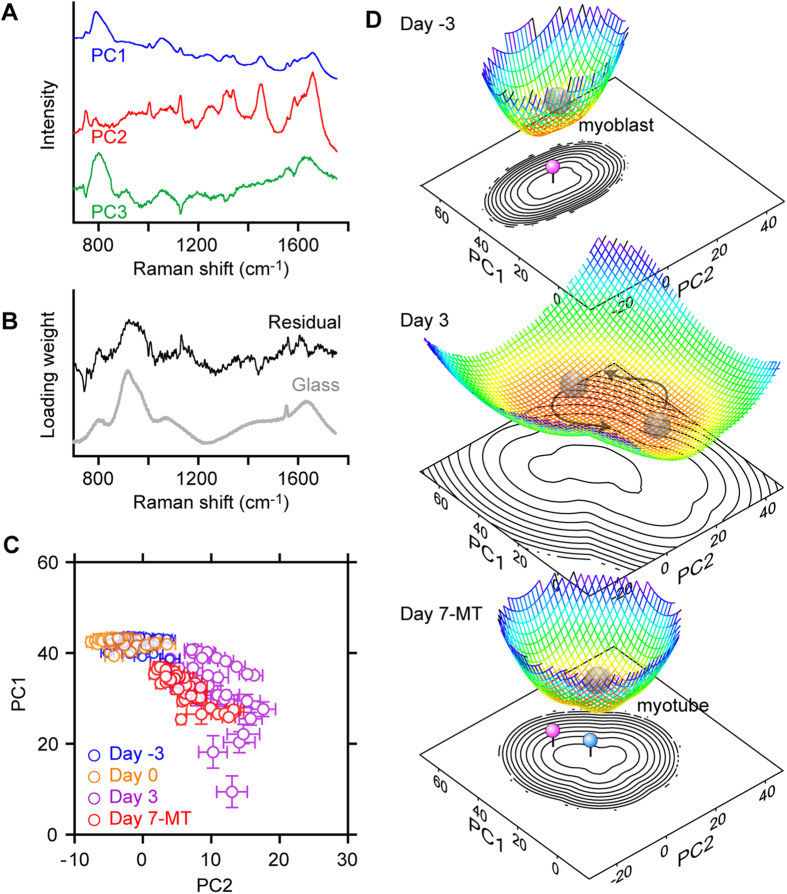
Alteration of the potential landscapes in C2C12 differentiation. (**A**) The first three PCA loading vectors calculated from Raman spectra of the nuclei at Day -3, Day 0 and Day 3. (**B**) Similarity between the averaged residual component of the Raman spectra of myotubes (Day 7-MT), which is given by subtraction of PC1–3 (black) and a Raman spectrum of a coverslip made of borosilicate glass (grey). (**C**) Plot of scores for PC1 and PC2. Each marker shows the average value of the scores for PC1 and PC2 of the Raman spectra obtained from single nuclei. Error bars show the SDs of the score values from the same nuclei. (**D**) Potential landscapes at Day -3, Day 3 and Day 7-MT on the PC1-PC2 plane, which were estimated from *U*(***t***) = −ln(*P*(***t***)). The counter plots corresponding to the potential landscapes are also shown. Cells in the homogeneous state at Day -3 started to show heterogeneity at Day 3 and became homogeneous again at Day 7. Note that the centre of the attractors for myoblasts and myotubes, indicated by pink and blue pins, respectively, are at different locations on the PC1-PC2 plane.

In the Raman observation of the four stages of C2C12 differentiation, there was a difference in sample configuration between the myotube sample (Day 7-MT) and the myoblast samples (Day -3, Day 0 and Day 3), which led to an obvious discrepancy of baseline shape in the Raman spectra (Fig. 2A,E). To circumvent this difference, we employed the following procedure for analysing the Raman spectra. First, we performed PCA against a dataset including the spectra of Day -3, Day 0 and Day 3 myoblasts to calculate loading vectors and PC scores for the cells. The loading vectors were used to construct the common state space. Then, the myotube spectra (Day 7-MT) were projected onto the state space by taking the inner product of each spectrum and the loading vectors. The inner product values correspond to the myotube scores. In this analysis, we only used the fingerprint region (700–1800 cm^−1^) of nuclear Raman spectra. Figure 3A shows the loading vectors for the first three PCs (PC1–3) calculated from the Raman spectra of myoblasts, which closely resemble the previously reported loading vectors calculated for ESCs^12^. Figure 3B shows the averaged spectrum of the residual components of the myotubes, which was calculated by subtraction of the three PCs from the original spectra. The residual spectrum is similar to the spectra from the glass coverslip (Fig. 3B, glass), indicating that decomposition of myotube Raman spectra successfully extracted the component from cells on a glass substrate.

We plotted the single cells for myotubes (Day 7-MT) and Day -3, Day 0 and Day 3 myoblasts on the two-dimensional coordinate system of the loading vectors (score plot, Fig. 3C and Fig. S3), with each dot corresponding to one nucleus and error bars representing the standard deviation (SD) of the score values within the nuclei. Sparsely cultured myoblasts (Day -3) clustered in the high-PC1/low-PC2 region in the PC1-PC2 plane, which was also true for cells that became confluent (Day 0); however, they were restricted to a slightly smaller region. On Day 3, cells were distributed in a wider area in the PC1-PC2 plane, and most cells occupied a different region than on Day -3 or Day 0. Further progression of differentiation led to formation of myotubes on Day 7, which appeared in a region different from that of myoblasts. This result indicates that differences in cell state appear as differences in Raman spectra, which is in good agreement with our previous report^12^. Thus, the cellular state transition during C2C12 differentiation could be visualized on a two-dimensional plane by using PCA.

The population distribution of cells in a specific state can be regarded to represent the relative depth of the potential landscape at that state; thus, by calculating the ratio in each cell state, the potential landscape can be estimated. The landscape of the energy-equivalent potential, *U*(***t***), can be expressed as a function of the experimentally obtained population density, *P*(***t***), in the equation *U*(***t***) = −(1/*D*)ln(*P*(***t***)), which is based on the well-known Boltzmann equation^18^. *D* denotes a hypothetical diffusion constant related to intrinsic fluctuation of the cellular state, and ***t*** denotes a set of PC scores used as state parameters. *D* is assumed to be a constant value so that it can be omitted because we are interested in the location and structure of attractors on the potential landscape. We can therefore re-define the potential in a further simple equation, *U*(***t***) = −ln(*P*(***t***))^4^. This method of interpreting population data was originally established in complex systems science^2^ and was applied to understanding the complex system of gene regulatory networks through both experimental and theoretical studies^3^,^4^,^19^. We here assume that, in place of gene expression level, the Raman spectrum is a state parameter for the coordinate axes of the state space for the potential landscape. In our dataset, the distributions of the PC scores are still discrete (Fig. 3C) because of the limited number of samples, but we wanted to construct a landscape with a smooth surface. It is preferable to have thousands of data points and use the raw population histogram to draw a smooth landscape, as other groups do with gene expression levels measured by flowcytometry^20^. We therefore employed the kernel density estimation method to estimate the population density function, *P*(***t***), from the discrete distribution^21^. We chose a Gaussian function as a kernel to prevent potential divergence to positive infinity. The PC score plots were convolved with a Gaussian function with a kernel width determined by Scott’s rule^21^.

The potential landscapes at the three stages of differentiation were drawn on a two-dimensional state space composed of the two state parameters, and the scores of PC1 and PC2 are shown in Fig. 3C. We successfully described the C2C12 differentiation landscape, clearly showing the disappearance and reappearance of the differentiation state attractor (Fig. 3D). The strong attractor present at Day -3 diminished at Day 3, indicating that cells can easily transition between states and that the cell population has a high degree of heterogeneity. Seven days after induction of differentiation (Day 7-MT), a strong attractor appeared in a location different from that of myoblasts (Day -3 and Day 3). Some cells in a state far from myotubes were probably eliminated by apoptosis, as cells entering apoptosis increased on Day 3 (Fig. S4). As mentioned, we established a procedure to draw a hypothetical attractor landscape using the Raman spectrum of the cell.

### Drawing the landscape of ESC differentiation

**Figure 4:**
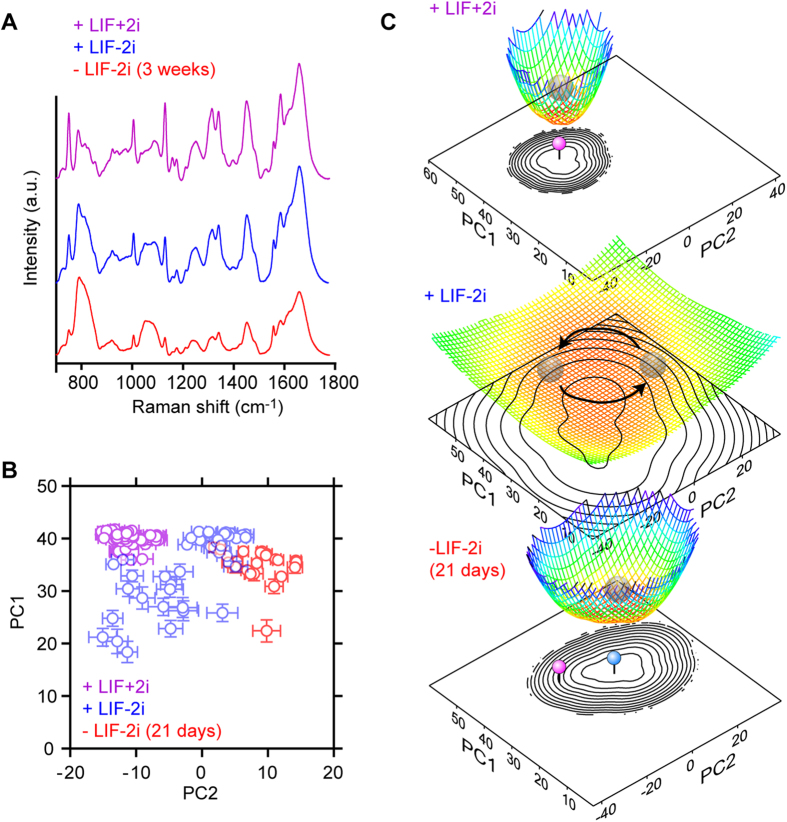
Alteration of the potential landscapes in ESC differentiation. (**A**) Averaged Raman spectra of ESCs in +LIF+2i culture (purple) +LIF-2i culture (blue) and -LIF-2i culture for 21 days (red). (**B**) Plot of scores for PC1 and PC2. Each marker shows the average value of the scores for PC1 and PC2 of the Raman spectra obtained from single nuclei. Error bars show the SDs of the score values from the same nuclei. Spectra were obtained from 62 nucleus for +LIF+2i, 44 for +LIF-2i and 22 for -LIF-2i condition. Data were obtained from three independent cell culture performed on different days. (**C**) Potential landscapes at the three conditions, which were estimated from *U*(***t***) = −ln(*P*(***t***)). The contour plots corresponding to the potential landscapes are also shown. The centres of the attractors indicated by the pink and blue pins are at different locations on the PC1-PC2 plane.

To elucidate whether disappearance of the strong attractor just before entering the differentiated state is a common phenomenon that can be seen in other cell types, we performed the same analysis for ESCs. We previously reported that mouse ESCs cultured in the presence of leukaemia inhibitory factor (LIF) show highly heterogeneous cell states, which became homogeneous after induction of differentiation^12^. Recently, several groups reported that undifferentiated mouse ESCs can be maintained in a more stable ground state when two small molecule inhibitors, mitogen-activated protein or extracellular signal-regulated kinase (MEK) inhibitor and glycogen synthase kinase 3 (GSK3) inhibitor (2i), are added to culture medium^22^,^23^. This indicates that ESCs cultured in the +LIF-2i condition are less undifferentiated than ESCs in the +LIF+2i condition, which are in a state just before entering differentiation. Therefore, we carried out Raman observation of ESCs in +LIF+2i, +LIF-2i and -LIF-2i conditions (Fig. S5). Spectra were obtained from 62 nucleus for +LIF+2i, 44 for +LIF-2i and 22 for -LIF-2i condition. Data was obtained from three independent cell culture performed on different days. In the -LIF-2i condition, Raman spectra were obtained 21 days after the removal of LIF and 2i to allow the cells to differentiate. Raman spectra showed large differences among the three conditions: peaks from cytochrome C were pronounced in the +LIF+2i condition (Fig. 4A), whereas spectra in the +LIF-2i and -LIF-2i conditions were similar to those reported previously^12^. We also found that the relative intensities of other peaks were slightly different. We performed PCA for all the spectral data in the three stages to calculate the loading vectors and scores for all the cells (Fig. S6). A PCA score plot (Fig. 4B and Fig. S7) showed that cell state in the +LIF+2i condition is relatively homogeneous and different from those in the +LIF-2i or -LIF-2i condition. ESCs in the +LIF-2i condition were heterogeneous and became homogeneous after differentiation, which is in good agreement with our finding for C2C12 cells. Based on this score plot, we drew a potential landscape of differentiation. In the estimation of the population density function, *P*(*t*), the outlier in the low-PC1/high-PC2 region was omitted to prevent a non-negligible modification of the structure of the landscape. The landscape clearly shows the disappearance of the strong attractor after 2i removal and reappearance of attractor after LIF removal (Fig. 4C), similar to C2C12 cells. It is important for stable cell culture that cell state does not easily fluctuate before induction of differentiation, thus the landscape should show deep attractor, and that this deep attractor must be weaken for cells to transit to different state at the onset of differentiation. Transition of the potential landscape structure of cell state visualized by Raman spectroscopy in C2C12 cells and ESC support this notion.

## Discussion

We successfully visualized the transition of the potential landscape structure, which features the disappearance and re-emergence of strong attractors during C2C12 differentiation, using Raman spectroscopy and PCA. We found that undifferentiated C2C12 myoblasts occupied a homogeneous cell state defined by Raman spectra (Day -3 and Day 0). Three days after induction of differentiation, the cell population showed highly heterogeneous cell states (Day 3), which became homogeneous again after formation of myotubes (Day 7-MT). The myotube attractor was identified at a different position than the original myoblast attractor. A similar result was obtained for ESCs, where cell state was less heterogeneous in the undifferentiated ground state and after differentiation. These results suggest the existence of a common attractor structure transition behaviour. In particular, the absence of a strong attractor at the early stage of differentiation seems to be an essential process for cell differentiation, where a cell stochastically changes its state before falling into an appropriate differentiated state. Cells with a strong attractor are stable and cannot easily proceed to a different cell state, and the strong attractor must therefore be weakened to change the cell state.

Cells were widely distributed in the PC1-PC2 plane of the score plot at Day 3 of C2C12 myogenesis, and a large cell population was observed in the region corresponding to neither myoblasts nor myotubes. The cell state occupied by this sub-population is of interest. Although immunofluorescence indicated heterogeneity in the cell state based on the expression levels of myogenin and MHC (Fig. S1C), this observation does not provide further information on the identity of cells in the sub-population, which do not express these proteins. In this regard, we found many cells floating on Day 3, indicating that many cells die at this time point (Fig. S4). Thus, some proportion of the sub-population visualized by Raman spectroscopy could be cells that are undergoing apoptosis. To evaluate this prediction, we performed a caspase 3/7 assay at various stages of C2C12 differentiation and found that apoptosis activity increased after induction of differentiation (Fig. S4). This result indicates that cells occupy various states after induction of differentiation, and only cells that went down the pathway leading to myotube formation survived.

In this study, cell state transitions during myogenesis and ESC differentiation were targeted as models of cell differentiation, both of which have been well studied and documented for the past several decades^24^,^25^,^26^. Most of these previous studies employed biochemical methods including western blot and immunohistochemistry, while Raman spectroscopy was used in this study. Despite the difficulty of making direct correlations between our results and others, we propose that cell state appearing in the PC1-PC2 plane of the score plot (Figs 3C and 4B) corresponds to the cell state revealed by the biochemical methods. Furthermore, our result strongly supports the effect of 2i on stable maintenance of undifferentiated ESCs, which is obvious in the comparison of the attractor structures for the +LIF+2i and +LIF-2i conditions. Further study is necessary to clarify the similarities and differences between information acquired by Raman spectroscopy and that of other conventional techniques.

In conclusion, we verified the ability of cellular Raman spectra to estimate the cell state in a non-invasive manner. Although there are many methods that can report changes in cell state, there is no other such method for analysis at the single-cell level or for analysing the cell state in a label-free and non-destructive manner. Thus, Raman spectroscopy can be a useful tool in determining the cell state. Future improvement of the throughput and temporal resolution of Raman measurements of cells will allow for estimation of more detailed potential landscape structures and the flow of cell state in the state space, respectively. The detailed map of state transition will contribute to the prediction and control of cell fate. However, unlike biochemical methods such as western blot analysis or real-time PCR, Raman spectroscopy does not provide information regarding the expression of a gene of interest, which makes interpretation difficult. To this end, Raman spectroscopy cannot be substituted for current biochemical methods but can complement them by non-invasively visualizing the cell state transition. By carefully matching the results obtained through biochemical analysis and Raman spectroscopy, detailed cell state information including gene expression can be estimated from Raman spectra alone.

## Methods

### Cell culture

C2C12 cells were purchased from the Riken Cell Bank (Ibaraki, Japan). The C2C12 cells were maintained in growth medium (GM) composed of Dulbecco’s modified Eagle’s medium (DMEM; Invitrogen, Carlsbad, CA) supplemented with 10% foetal bovine serum (FBS; Sigma–Aldrich, St. Louis, MO) and antibiotics (100 U/mL potassium penicillin G and 0.1 mg/mL streptomycin (Invitrogen)). The cells were passaged before reaching confluence and used within ten passages. To induce differentiation, cells were cultured until confluence and differentiated in DMEM supplemented with 2% calf serum (Sigma–Aldrich) and antibiotics. Medium was exchanged every day. Mouse ESCs (E14Tg2a) were maintained on feeder-free gelatine-coated plates in LIF-supplemented medium: DMEM-High Glucose (DMEM-HG; Invitrogen, Carlsbad, CA) was supplemented with 10% FBS (Invitrogen), antibiotics (100 U/mL penicillin and 0.1 mg/mL streptomycin) (Invitrogen), 1 × GlutaMAX-1 (Invitrogen), 1% 2-mercaptoethanol (Bio-Rad), 1 × nonessential amino acids (Invitrogen), and 1 mM sodium pyruvate (Sigma-Aldrich).

### Western blot analysis

The cells were solubilized in a solution containing 5% v/v sodium dodecyl sulphate (SDS), 5% v/v 2-mercaptoethanol, 10% v/v glycerol, 0.0033% bromophenol blue and 125 mM Tris-HCl (pH 6.8), sonicated for 1 min, then boiled at 90 °C for 3 min. Protein concentrations were determined using the Pyrogallol Red-Molybdate assay (Wako Chemicals, Miyazaki, Japan) and normalized before loading. SDS-polyacrylamide gel electrophoresis was carried on 5% stacking and 12% running gels. The proteins were transferred to a polyvinylidene difluoride (PVDF) membrane (Immobilon-P; Millipore, Bedford, MA), and the membrane was blocked with 5% non-fat dry milk in Tris-buffered saline containing 0.1% Tween 20 (TBS-tw) for 1 h at room temperature. Immunoblotting to detect individual proteins was achieved by 1 h incubation with a 1:1000 dilution of antibodies against myogenin (ab1835; abcam Inc., Cambridge, MA), MHC (sc20641; Santa Cruz Biotechnology, Santa Cruz, CA), and tropomyosin (ab7785; abcam) in 1% bovine serum albumin/TBS-tw. Then, incubation with a 1:5000 dilution of horseradish peroxidase-conjugated secondary antibodies (Pierce, Rockford, IL) was carried out for 45 min at room temperature. A SuperSignal Chemiluminescence detection kit (Pierce, Rockford, IL) was used according to the manufacturer’s instructions.

### Microscopy

For Raman imaging, cells were seeded on a silica coverslip (SPI supplies, West Chester, PA) coated with 0.1% gelatine and cultured for three days. To observe myotubes, cells were cultured on a borosilicate glass (BK7) coverslip coated with 0.1% gelatine. Immediately before observation, the medium was replaced with Tyrode’s solution. Cells on a BK7 coverslip were placed on a silica coverslip cell-side down so that the cells were attached to the silica coverslip. All data were recorded with a custom-built slit-scanning Raman microscope based on an inverted microscope with an excitation wavelength of 532 nm for all observations^27^. A NIKON CFI Plan Apo IR 60 × water immersion lens with a 1.27 numerical aperture (NA) was used as the objective lens. The Perfect Focus System remained active during all measurements. The images were scanned over a 40 μm range and divided into 120 lines. The exposure time for each line was 5 s with 2.4 mW/μm^2^ laser intensity.

### Peak size calculation

For the calculation of the sizes of the Raman peaks, we employed two distinct methods depending on the peak shape. For the peaks which are well isolated from the surrounding peaks, including peaks at 1310 cm^−1^, 1340 cm^−1^, 1450 cm^−1^, 1580 cm^−1^ and 1660 cm^−1^, Lorentzian curve fitting with a linear baseline was employed. Neighbouring two peaks (1310 cm^−1^ and 1340 cm^−1^, 1580 cm^−1^ and 1660 cm^−1^) were simultaneously calculated. For the peaks which are located either on a slope of a broad peak or at the valley between two larger peaks, including 1004 cm^−1^, 1127 cm^−1^, 1176 cm^−1^ and 1396 cm^−1^, the Covell method was employed^28^.

### Data analysis

For spectral characterization, PCA was employed using a non-linear iterative partial least squares (NIPALS) algorithm as reported previously^29^. As a pre-treatment for PCA, we standardized the original spectral data by subtracting the mean value from each spectrum and dividing by its SD^29^. The standardization process is effective at eliminating both the additive and multiplicative differences of the spectral baseline caused by slight discrepancies in the experimental conditions.

### Statistical analysis

The paired t-test was used to analyse the data. A difference with a value of P < 0.05 as compared to control values was considered to be statistically significant and indicated by asterisks.

## Additional Information

**How to cite this article**: Ichimura, T. *et al.* Visualizing the appearance and disappearance of the attractor of differentiation using Raman spectral imaging. *Sci. Rep.* **5**, 11358; doi: 10.1038/srep11358 (2015).

## Supplementary Material

Supplementary Information
