# Supplementary material for: Visualizing the appearance and disappearance of the attractor of differentiation using Raman spectral imaging
Source: Sci Rep. 2015 Jun 16;5:11358. doi: 10.1038/srep11358 (PMC5155549; doi:10.1038/srep11358)
Supplement: Supplementary Information [file srep11358-s1.doc]

**Supplementary Information:**

Visualizing the appearance and disappearance of the attractor of differentiation using Raman spectral imaging

Taro Ichimura^1^, Liang-da Chiu^2^, Katsumasa Fujita^2^, Hiroaki Machiyama^3^, Satoshi Kawata^2,4^, Tomonobu M. Watanabe^1^, and Hideaki Fujita^1,3*^

^1^*Laboratory for Comprehensive Bioimaging, Riken QBiC, 6-2-3 Furuedai, Suita, Osaka, Japan*

^2^*Department of Applied Physics, Osaka University, 2-1 Yamadaoka, Suita, Osaka, Japan*

*^3^WPI, Immunology Frontier Research Center, Osaka University, 1-3 Yamadaoka, Suita, Osaka, Japan*

*^4^Nanophotonics Laboratory, RIKEN, Wako, Saitama, Japan, 2-1 Hirosawa, Wako, Saitama, Japan*

Short title: Raman imaging of differentiation

Keywords: C2C12; muscle; attractor; spectroscopy; differentiation

*Corresponding author: Hideaki Fujita, Ph.D., Laboratory for Comprehensive Bioimaging, Riken Quantitative Biology Center, OLABB, 6-2-3 Furuedai, Suita, Osaka 565-0874, Japan. Tel: +81-6-6155-0111. Fax: +81-6-6155-0112. E-mail: hideaki.fujita@riken.jp

**
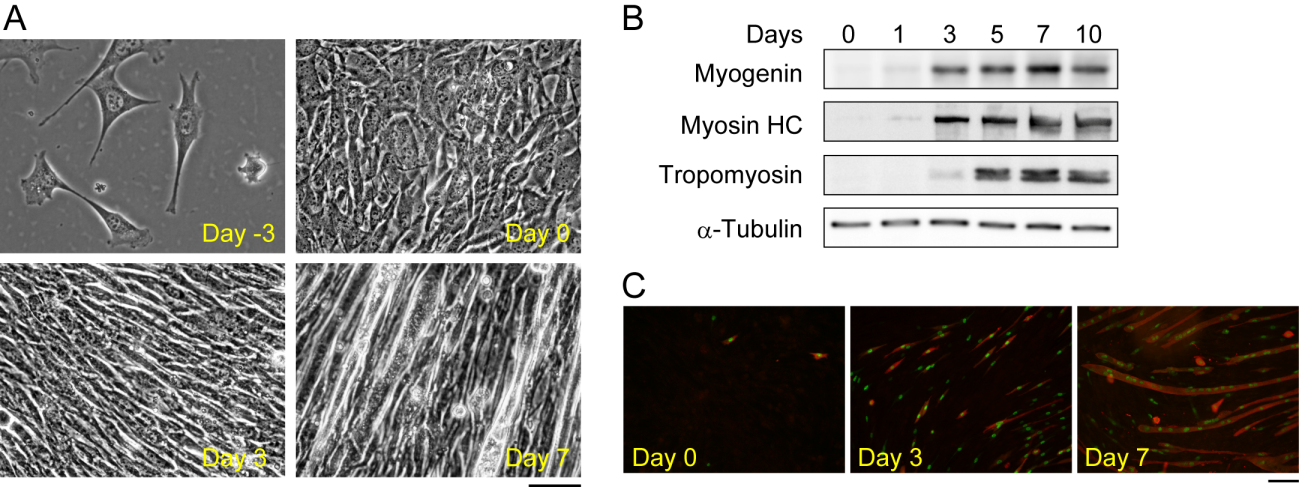
**

**Figure S1.** Differentiation of C2C12 myoblasts. (A) Phase contrast images of C2C12 myoblasts sparsely cultured (Day -3), being confluent (Day 0), 3 days after induction of differentiation (Day 3), and 7 days after induction of differentiation (Day 7). Scale bar, 50 μm. Western blot analysis against myogenin, myosin heavy chain (Myosin HC), tropomyosin, and α-tubulin (α-Tubulin) at various stages of differentiation. Days denote days after induction of differentiation. (C) Immuno-fluorescent micrograph of C2C12 myoblasts when cells are confluent (Day 0), 3 days after induction of differentiation (Day 3), and 7 days after induction of differentiation (Day 7). Cells were stained against anti-myogenin (green) and anti-myosin heavy chain (red) antibodies. Scale bar, 100 μm.


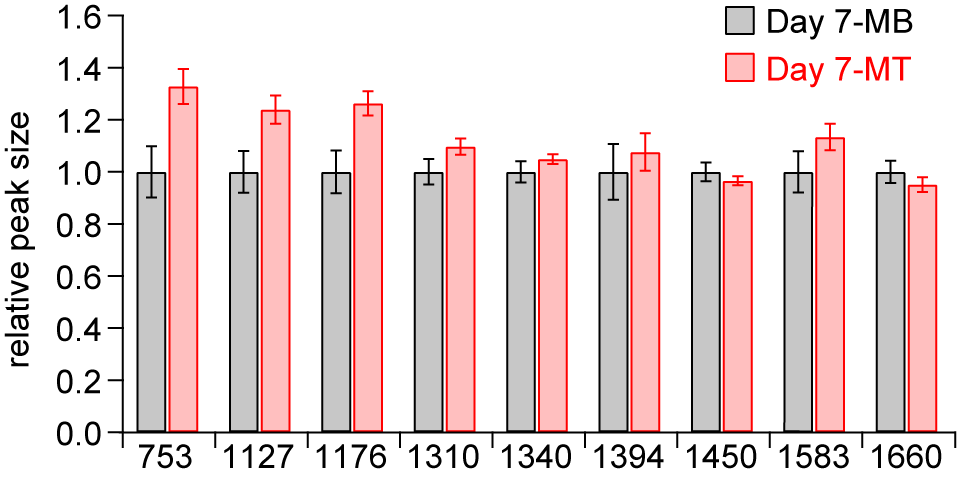


**Figure S2.** Relative area of several Raman peaks of myoblasts (Day 7-MB) and myotubes (Day 7-MT). Each peak area was normalized to the peak area of 1004 cm^-1^. Data are displayed relative to myoblast. Scale bar, SE.


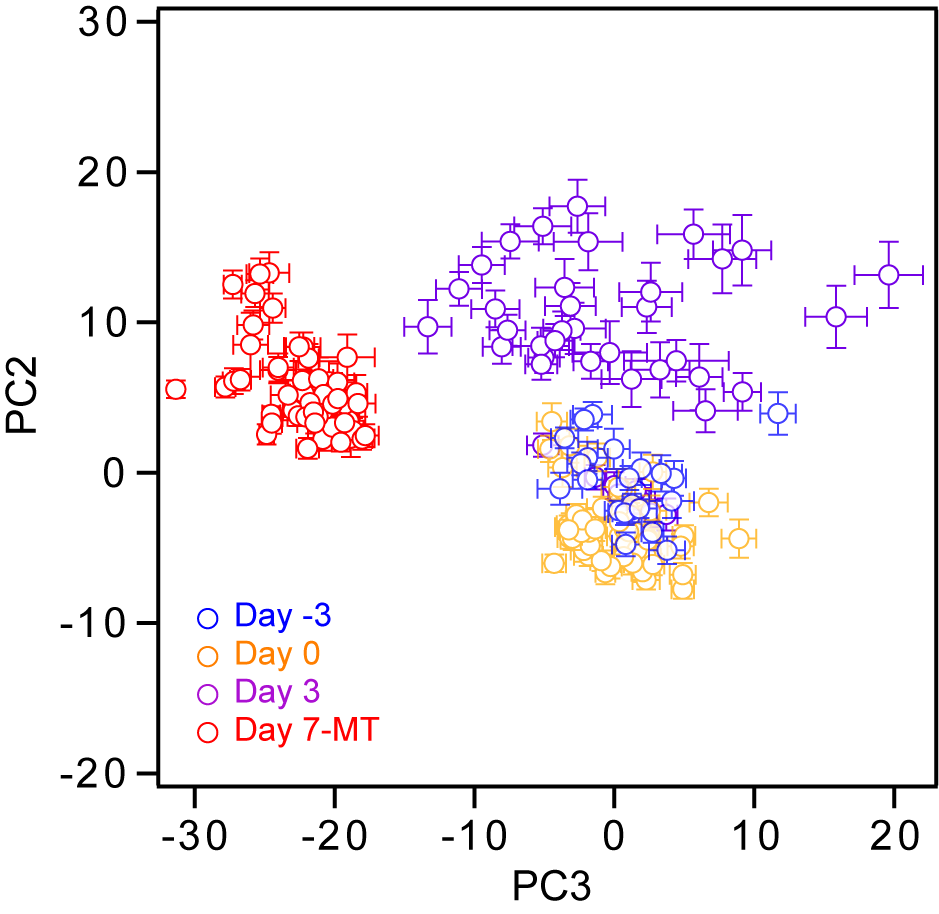


**Figure S3.** Score plot of PC3 vs PC2 for Day -3, Day 0, Day 3 myoblasts and myotubes. Each marker shows the average value of the scores for PC3 and PC2 of the Raman spectra obtained from single nuclei. Error bars show the SDs of the score values from the same nuclei.

**Figure S4.** Phase contrast images of C2C12 cells showing the increase in dead cells. (A) Cells just before induction of differentiation. (B) Three days after induction of differentiation. Medium was changed 24 h before observation. Scale bar, 200 μm. (C, D) Caspase 3/7 activity in C2C12 cells before and after induction of differentiation. (C) Cells just after becoming confluent. (D) Three days after induction of differentiation. Cells were stained against caspase 3/4 (green) and with propidium iodide (red). Scale bar, 100 μm.

**Figure S5.** Raman images of ESC in various culture conditions. (A) ESC cultured in +LIF+2i condition. (B) ESC cultured in +LIF-2i condition. (C) ESC cultured in -LIF-2i condition for 3 weeks. Scale bar, 10 μm.

**Figure S6.** The first three PCA loading vectors calculated from Raman spectra of the ESC nuclei at +LIF+2i, +LIF-2i and -LIF-2i.

**Figure S7.** Score plot of PC3 vs PC2 for ESC cultured in +LIF+2i, +LIF-2i and -LIF-2i conditions. Each marker shows the average value of the scores for PC3 and PC2 of the Raman spectra obtained from single nuclei. Error bars show the SDs of the score values from the same nuclei.

**Table S1: Percentage of variance**

| **Cell Type** | **PC1** | **PC2** | **PC3** | **PC4** | **PC5** |
| --- | --- | --- | --- | --- | --- |
| **C2C12** | 91.33 | 4.86 | 2.94 | 0.48 | 0.40 |
| **ESC** | 93.01 | 3.66 | 2.53 | 0.41 | 0.18 |
